# A triclinic modification of 3,4-dihy­droxy­benzoic acid monohydrate

**DOI:** 10.1107/S1600536811034635

**Published:** 2011-08-27

**Authors:** Seik Weng Ng

**Affiliations:** a Department of Chemistry, University of Malaya, 50603 Kuala Lumpur, Malaysia, and Chemistry Department, Faculty of Science, King Abdulaziz University, PO Box 80203 Jeddah, Saudi Arabia

## Abstract

The unit cell of the title compound, C_7_H_6_O_4_·H_2_O, features four independent formula units; the individual carb­oxy­lic acid mol­ecules themselves are nearly planar (r.m.s. deviations = 0.0189, 0.0334, 0.0356 and 0.0441 Å). Two independent mol­ecules each form two hydrogen bonds by acid–carbonyl O—H⋯O inter­actions and the dimers are also nearly planar (r.m.s. deviations = 0.039 and 0.049 Å). The two independent dimers are aligned at 44.5 (1)°. Other O—H⋯O inter­actions involving the hy­droxy groups and water mol­ecules give rise to a three-dimensional network.

## Related literature

For the triclinic modification whose cell is about half the volume of the present triclinic modification, see: Horneffer *et al.* (1999 ▶).
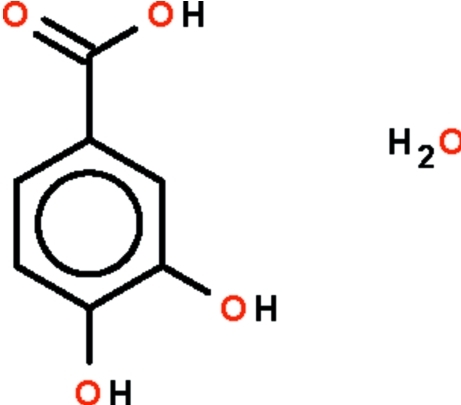


## Experimental

### 

#### Crystal data

C_7_H_6_O_4_·H_2_O
*M* *_r_* = 172.13
Triclinic, 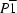

*a* = 7.1105 (3) Å
*b* = 12.7807 (5) Å
*c* = 17.5318 (7) Å
α = 72.491 (4)°
β = 89.901 (3)°
γ = 74.457 (3)°
*V* = 1458.45 (10) Å^3^
*Z* = 8
Cu *K*α radiation
μ = 1.18 mm^−1^
*T* = 100 K
0.20 × 0.10 × 0.10 mm

#### Data collection

Agilent Technologies SuperNova Dual diffractometer with Atlas detector
Absorption correction: multi-scan (*CrysAlis PRO*; Agilent, 2010 ▶) *T*_min_ = 0.798, *T*_max_ = 0.891
9167 measured reflections
5301 independent reflections
4910 reflections with *I* > 2σ(*I*)
*R*_int_ = 0.020

#### Refinement

*R*[*F* ^2^ > 2σ(*F* ^2^)] = 0.056
*wR*(*F* ^2^) = 0.161
*S* = 1.15
5301 reflections
513 parameters
24 restraints
H atoms treated by a mixture of independent and constrained refinement
Δρ_max_ = 0.31 e Å^−3^
Δρ_min_ = −0.35 e Å^−3^

### 

Data collection: *CrysAlis PRO* (Agilent, 2010 ▶); cell refinement: *CrysAlis PRO*; data reduction: *CrysAlis PRO*; program(s) used to solve structure: *SHELXS97* (Sheldrick, 2008 ▶); program(s) used to refine structure: *SHELXL97* (Sheldrick, 2008 ▶); molecular graphics: *X-SEED* (Barbour, 2001 ▶); software used to prepare material for publication: *publCIF* (Westrip, 2010 ▶).

## Supplementary Material

Crystal structure: contains datablock(s) global, I. DOI: 10.1107/S1600536811034635/xu5306sup1.cif

Structure factors: contains datablock(s) I. DOI: 10.1107/S1600536811034635/xu5306Isup2.hkl

Supplementary material file. DOI: 10.1107/S1600536811034635/xu5306Isup3.cml

Additional supplementary materials:  crystallographic information; 3D view; checkCIF report

## Figures and Tables

**Table 1:** Hydrogen-bond geometry (Å, °)

*D*—H⋯*A*	*D*—H	H⋯*A*	*D*⋯*A*	*D*—H⋯*A*
O1—H1⋯O6	0.85 (1)	1.83 (1)	2.678 (3)	178 (3)
O3—H3⋯O1*W*^i^	0.84 (1)	1.99 (1)	2.803 (3)	163 (3)
O4—H4⋯O4*W*^ii^	0.84 (1)	1.86 (2)	2.674 (3)	163 (4)
O5—H5⋯O2	0.85 (1)	1.76 (1)	2.593 (3)	170 (4)
O7—H7⋯O3^iii^	0.84 (1)	1.91 (1)	2.746 (3)	172 (4)
O8—H8⋯O3*W*^iv^	0.84 (1)	1.94 (1)	2.772 (3)	171 (4)
O9—H9⋯O14	0.84 (1)	1.84 (2)	2.664 (3)	166 (5)
O11—H11⋯O3*W*^v^	0.84 (1)	2.02 (2)	2.824 (3)	161 (4)
O12—H12⋯O1*W*	0.84 (1)	1.92 (1)	2.760 (3)	175 (4)
O13—H13⋯O10	0.84 (1)	1.76 (1)	2.599 (3)	171 (5)
O15—H15⋯O11^vi^	0.84 (1)	1.91 (1)	2.754 (2)	178 (4)
O16—H16⋯O2*W*^vii^	0.84 (1)	1.92 (2)	2.699 (3)	153 (4)
O1*W*—H1*W*1⋯O2	0.84 (1)	2.36 (5)	2.920 (3)	124 (4)
O1*W*—H1*W*2⋯O2*W*^viii^	0.84 (1)	2.07 (1)	2.911 (4)	173 (7)
O2*W*—H2*W*1⋯O6	0.85 (1)	2.24 (1)	3.071 (3)	168 (4)
O2*W*—H2*W*2⋯O7^ix^	0.85 (1)	2.00 (2)	2.823 (3)	165 (4)
O3*W*—H3*W*1⋯O10	0.85 (1)	2.40 (4)	2.933 (3)	122 (3)
O3*W*—H3*W*2⋯O4*W*^x^	0.84 (1)	2.08 (1)	2.920 (4)	174 (6)
O4*W*—H4*W*1⋯O14	0.85 (1)	2.30 (3)	3.079 (3)	152 (5)
O4*W*—H4*W*2⋯O15^xi^	0.86 (1)	1.97 (1)	2.806 (3)	164 (4)

Symmetry codes: (i) 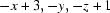; (ii) 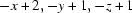; (iii) 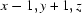; (iv) 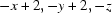; (v) 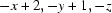; (vi) 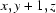; (vii) 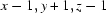; (viii) 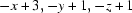; (ix) 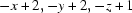; (x) 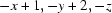; (xi) 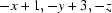.

**Fig. 1.**
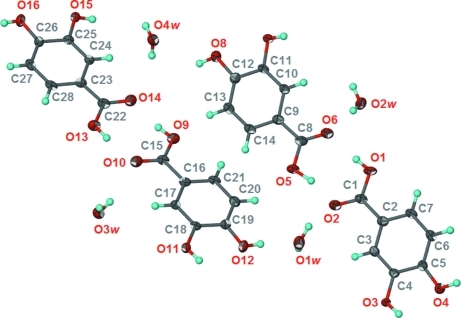
Thermal ellipsoid plot (Barbour, 2001) of the four independent formula units molecules of C7H6O4.H2O at the 70% probability level; hydrogen atoms are drawn as spheres of arbitrary radius.

## supplementary crystallographic information

### Comment

3,4-Dihydroxybenzoic acid monohydrate (Scheme I) was reported as a triclinic crystal [*a* 8.045, *b* 8.134, *c* 12.692 Å; *α* 71.58, *β* 76.79, γ 72.17 °]; there are two independent formula units in the unit cell (Horneffer *et al.*, 1999). The carboxylic acid is a commerically available compound. This carboxylic acid when isolated from a plant crystallizes as a monohydrate and its unit cell is twice as large so that there are four independent molecules (Fig. 1). The individual carboxylic acid molecules themselves are planar. Two independent molecules form two hydrogen bonds by *O*–H_acid_···O_carbonyl_ interactions. Other O–H···O interactions that involve the hydroxy groups and water molecules give rise to a three-dimensional network (Table 1).

### Experimental

The aerial part of D*odonaea viscosa* (20 kg) was powdered and extracted with methanol (10 *L x* 3) at room temperature and the crude residue (2 kg) was obtained after removal of methanol under reduced pressure. The residue was suspended in water and extracted with *n-*hexane,chloroform, ethyl acetate and *n*-butanol. The ethyl acetate fraction (250 g) was subjected repeatedly to column chromatography on silica gel. The compound (10 mg) was found in a polarity range 50% ethyl acetate to 50% ethyl acetate:*n*-hexane. Crystals of 3,4-dihydroxybenzoic acid monohydrate were the unexpected compound that were obtained by recrystallization from a 1:1 acetone:*n*-hexane mixture.

### Refinement

Carbon-bound H-atoms were placed in calculated positions [C—H 0.95 Å, *U*_iso_(H) 1.2*U*_eq_(C)] and were included in the refinement in the riding model approximation.

The hydroxy and water H-atoms were located in a difference Fourier map, and were refined with distance restraints of O–H 0.84±0.01 and H···H 1.37±0.01 Å; their temperature factors were refined.

Although the crystal was measured to a 2θ limit of 150 °, the reflections beyond 140 ° were omitted. Also omitted from the refinement were (-1 - 6 10), (1 - 4 12), (-1 5 15) and (1 6 14).

### Crystal data

**Table:**

C_7_H_6_O_4_·H_2_O	*Z* = 8
*M**_r_* = 172.13	*F*(000) = 720
Triclinic, *P*1	*D*_x_ = 1.568 Mg m^−^^3^
Hall symbol: -P 1	Cu *K*α radiation, λ = 1.54184 Å
*a* = 7.1105 (3) Å	Cell parameters from 5539 reflections
*b* = 12.7807 (5) Å	θ = 2.7–74.2°
*c* = 17.5318 (7) Å	µ = 1.18 mm^−^^1^
α = 72.491 (4)°	*T* = 100 K
β = 89.901 (3)°	Block, colorless
γ = 74.457 (3)°	0.20 × 0.10 × 0.10 mm
*V* = 1458.45 (10) Å^3^	

### Data collection

**Table:**

Agilent Technologies SuperNova Dual diffractometer with Atlas detector	5301 independent reflections
Radiation source: SuperNova (Cu) X-ray Source	4910 reflections with *I* > 2σ(*I*)
Mirror	*R*_int_ = 0.020
Detector resolution: 10.4041 pixels mm^-1^	θ_max_ = 70.0°, θ_min_ = 2.7°
ω scan	*h* = −8→8
Absorption correction: multi-scan (*CrysAlis PRO*; Agilent, 2010)	*k* = −12→15
*T*_min_ = 0.798, *T*_max_ = 0.891	*l* = −19→21
9167 measured reflections	

### Refinement

**Table:**

Refinement on *F*^2^	Primary atom site location: structure-invariant direct methods
Least-squares matrix: full	Secondary atom site location: difference Fourier map
*R*[*F*^2^ > 2σ(*F*^2^)] = 0.056	Hydrogen site location: inferred from neighbouring sites
*wR*(*F*^2^) = 0.161	H atoms treated by a mixture of independent and constrained refinement
*S* = 1.15	*w* = 1/[σ^2^(*F*_o_^2^) + (0.0556*P*)^2^ + 2.5625*P*] where *P* = (*F*_o_^2^ + 2*F*_c_^2^)/3
5301 reflections	(Δ/σ)_max_ = 0.001
513 parameters	Δρ_max_ = 0.31 e Å^−^^3^
24 restraints	Δρ_min_ = −0.35 e Å^−^^3^

### Fractional atomic coordinates and isotropic or equivalent isotropic displacement parameters (Å^2^)

**Table:**

	*x*	*y*	*z*	*U*_iso_*/*U*_eq_	
O1	1.2471 (3)	0.40038 (16)	0.61115 (12)	0.0256 (4)	
O2	1.3653 (3)	0.37601 (17)	0.49778 (12)	0.0300 (5)	
O3	1.6748 (3)	−0.04656 (15)	0.56841 (10)	0.0180 (4)	
O4	1.5861 (3)	−0.13742 (16)	0.71721 (11)	0.0252 (4)	
O5	1.2564 (3)	0.58919 (16)	0.41063 (12)	0.0251 (4)	
O6	1.1385 (3)	0.62121 (16)	0.52267 (12)	0.0284 (5)	
O7	0.8230 (3)	1.05257 (15)	0.42888 (11)	0.0195 (4)	
O8	0.9107 (3)	1.12551 (16)	0.28104 (11)	0.0248 (4)	
O9	0.8607 (3)	0.89145 (17)	0.11507 (12)	0.0253 (4)	
O10	0.7645 (3)	0.86456 (16)	0.00291 (12)	0.0284 (5)	
O11	0.8783 (3)	0.44030 (15)	0.07687 (10)	0.0187 (4)	
O12	1.0448 (3)	0.35333 (16)	0.22821 (11)	0.0236 (4)	
O13	0.6605 (3)	1.07815 (17)	−0.08454 (12)	0.0249 (4)	
O14	0.7424 (3)	1.11112 (16)	0.02742 (12)	0.0289 (5)	
O15	0.6378 (3)	1.54151 (15)	−0.06371 (10)	0.0189 (4)	
O16	0.4748 (3)	1.61621 (16)	−0.21018 (11)	0.0249 (4)	
O1w	1.2446 (4)	0.28526 (18)	0.37766 (13)	0.0341 (5)	
O2w	1.3495 (4)	0.70908 (18)	0.63249 (12)	0.0331 (5)	
O3w	0.9674 (4)	0.79992 (18)	−0.12887 (12)	0.0338 (5)	
O4w	0.4321 (4)	1.22050 (17)	0.12445 (12)	0.0330 (5)	
C1	1.3388 (4)	0.3346 (2)	0.56934 (16)	0.0203 (5)	
C2	1.4042 (4)	0.2126 (2)	0.61156 (15)	0.0177 (5)	
C3	1.5080 (3)	0.1395 (2)	0.57062 (15)	0.0165 (5)	
H3A	1.5360	0.1702	0.5168	0.020*	
C4	1.5690 (3)	0.0240 (2)	0.60809 (15)	0.0157 (5)	
C5	1.5253 (4)	−0.0231 (2)	0.68730 (15)	0.0176 (5)	
C6	1.4231 (4)	0.0501 (2)	0.72805 (15)	0.0195 (5)	
H6	1.3942	0.0193	0.7817	0.023*	
C7	1.3637 (4)	0.1667 (2)	0.69096 (15)	0.0190 (5)	
H7A	1.2953	0.2159	0.7193	0.023*	
C8	1.1644 (4)	0.6581 (2)	0.45019 (16)	0.0205 (5)	
C9	1.0988 (4)	0.7786 (2)	0.40319 (15)	0.0180 (5)	
C10	0.9914 (4)	0.8576 (2)	0.43934 (15)	0.0168 (5)	
H10	0.9615	0.8316	0.4934	0.020*	
C11	0.9296 (4)	0.9715 (2)	0.39720 (15)	0.0169 (5)	
C12	0.9758 (4)	1.0115 (2)	0.31738 (15)	0.0183 (5)	
C13	1.0818 (4)	0.9330 (2)	0.28172 (15)	0.0195 (5)	
H13A	1.1135	0.9590	0.2279	0.023*	
C14	1.1415 (4)	0.8177 (2)	0.32373 (15)	0.0185 (5)	
H14	1.2118	0.7648	0.2984	0.022*	
C15	0.8338 (4)	0.8252 (2)	0.07383 (16)	0.0204 (5)	
C16	0.8902 (4)	0.7023 (2)	0.11704 (15)	0.0180 (5)	
C17	0.8602 (3)	0.6279 (2)	0.07704 (15)	0.0163 (5)	
H17A	0.8046	0.6572	0.0230	0.020*	
C18	0.9116 (4)	0.5119 (2)	0.11619 (15)	0.0165 (5)	
C19	0.9973 (4)	0.4682 (2)	0.19596 (15)	0.0179 (5)	
C20	1.0265 (4)	0.5431 (2)	0.23495 (15)	0.0198 (5)	
H20	1.0834	0.5141	0.2888	0.024*	
C21	0.9735 (4)	0.6593 (2)	0.19609 (16)	0.0190 (5)	
H21	0.9939	0.7100	0.2232	0.023*	
C22	0.6821 (4)	1.1473 (2)	−0.04506 (16)	0.0213 (6)	
C23	0.6298 (4)	1.2688 (2)	−0.09188 (15)	0.0181 (5)	
C24	0.6587 (4)	1.3467 (2)	−0.05472 (15)	0.0170 (5)	
H24A	0.7125	1.3200	−0.0005	0.020*	
C25	0.6095 (4)	1.4616 (2)	−0.09623 (15)	0.0166 (5)	
C26	0.5238 (4)	1.5018 (2)	−0.17551 (15)	0.0172 (5)	
C27	0.4966 (4)	1.4243 (2)	−0.21265 (15)	0.0196 (5)	
H27	0.4411	1.4510	−0.2666	0.023*	
C28	0.5500 (4)	1.3081 (2)	−0.17132 (16)	0.0191 (5)	
H28	0.5322	1.2554	−0.1971	0.023*	
H1	1.212 (5)	0.4696 (12)	0.5821 (17)	0.030 (9)*	
H3	1.694 (5)	−0.1161 (11)	0.5938 (18)	0.031 (9)*	
H4	1.557 (6)	−0.162 (3)	0.7646 (10)	0.051 (12)*	
H5	1.289 (5)	0.5224 (15)	0.4440 (17)	0.039 (10)*	
H7	0.781 (6)	1.016 (3)	0.4706 (16)	0.065 (14)*	
H8	0.942 (6)	1.143 (4)	0.2336 (11)	0.062 (13)*	
H9	0.811 (7)	0.9575 (19)	0.083 (2)	0.078 (16)*	
H11	0.916 (5)	0.3703 (11)	0.1026 (19)	0.038 (10)*	
H12	1.100 (5)	0.335 (3)	0.2745 (11)	0.045 (11)*	
H13	0.689 (6)	1.0076 (12)	−0.060 (2)	0.061 (14)*	
H15	0.710 (5)	1.511 (3)	−0.0201 (13)	0.049 (12)*	
H16	0.416 (6)	1.629 (4)	−0.2551 (13)	0.054 (12)*	
H1w1	1.193 (7)	0.315 (5)	0.412 (3)	0.12 (2)*	
H1w2	1.358 (4)	0.293 (6)	0.372 (4)	0.13 (3)*	
H2w1	1.280 (5)	0.683 (3)	0.608 (2)	0.055 (13)*	
H2w2	1.293 (5)	0.7797 (11)	0.623 (2)	0.046 (11)*	
H3w1	0.994 (6)	0.825 (4)	−0.092 (2)	0.073 (16)*	
H3w2	0.855 (4)	0.791 (5)	−0.124 (3)	0.12 (3)*	
H4w1	0.544 (4)	1.189 (3)	0.112 (3)	0.091 (19)*	
H4w2	0.425 (5)	1.2913 (11)	0.114 (2)	0.045 (11)*	

### Atomic displacement parameters (Å^2^)

**Table:**

	*U*^11^	*U*^22^	*U*^33^	*U*^12^	*U*^13^	*U*^23^
O1	0.0350 (11)	0.0155 (11)	0.0266 (10)	−0.0025 (8)	0.0079 (8)	−0.0112 (8)
O2	0.0478 (12)	0.0167 (10)	0.0235 (10)	−0.0033 (9)	0.0084 (9)	−0.0084 (8)
O3	0.0251 (9)	0.0119 (10)	0.0149 (9)	−0.0010 (7)	0.0031 (7)	−0.0049 (7)
O4	0.0350 (11)	0.0174 (10)	0.0181 (10)	−0.0018 (8)	0.0062 (8)	−0.0030 (8)
O5	0.0351 (11)	0.0157 (11)	0.0237 (10)	−0.0015 (8)	0.0047 (8)	−0.0099 (8)
O6	0.0437 (12)	0.0168 (10)	0.0246 (10)	−0.0057 (9)	0.0097 (9)	−0.0087 (8)
O7	0.0269 (10)	0.0138 (9)	0.0170 (9)	−0.0024 (7)	0.0049 (7)	−0.0064 (7)
O8	0.0351 (11)	0.0176 (10)	0.0174 (9)	−0.0043 (8)	0.0057 (8)	−0.0024 (8)
O9	0.0362 (11)	0.0163 (11)	0.0247 (10)	−0.0057 (8)	−0.0017 (8)	−0.0099 (8)
O10	0.0464 (12)	0.0152 (10)	0.0230 (10)	−0.0049 (9)	−0.0029 (9)	−0.0081 (8)
O11	0.0292 (10)	0.0120 (10)	0.0146 (9)	−0.0049 (7)	−0.0020 (7)	−0.0046 (7)
O12	0.0348 (11)	0.0158 (10)	0.0168 (9)	−0.0042 (8)	−0.0055 (8)	−0.0027 (7)
O13	0.0368 (11)	0.0161 (11)	0.0245 (10)	−0.0074 (8)	0.0012 (8)	−0.0102 (8)
O14	0.0463 (12)	0.0154 (10)	0.0229 (10)	−0.0037 (9)	−0.0040 (9)	−0.0075 (8)
O15	0.0281 (10)	0.0131 (9)	0.0147 (9)	−0.0041 (7)	−0.0014 (7)	−0.0046 (7)
O16	0.0376 (11)	0.0175 (10)	0.0162 (9)	−0.0053 (8)	−0.0043 (8)	−0.0023 (7)
O1w	0.0593 (15)	0.0198 (11)	0.0204 (10)	−0.0069 (10)	−0.0070 (10)	−0.0061 (8)
O2w	0.0565 (14)	0.0154 (11)	0.0212 (10)	−0.0006 (10)	−0.0084 (9)	−0.0053 (8)
O3w	0.0538 (15)	0.0214 (11)	0.0203 (10)	−0.0014 (10)	0.0076 (9)	−0.0057 (8)
O4w	0.0621 (15)	0.0156 (11)	0.0192 (10)	−0.0077 (10)	0.0083 (10)	−0.0053 (8)
C1	0.0218 (12)	0.0198 (14)	0.0232 (13)	−0.0056 (10)	0.0034 (10)	−0.0126 (11)
C2	0.0175 (12)	0.0183 (14)	0.0195 (12)	−0.0052 (10)	0.0002 (9)	−0.0090 (10)
C3	0.0181 (12)	0.0179 (14)	0.0154 (12)	−0.0061 (10)	0.0007 (9)	−0.0070 (10)
C4	0.0171 (11)	0.0158 (13)	0.0159 (12)	−0.0038 (9)	0.0011 (9)	−0.0080 (10)
C5	0.0198 (12)	0.0175 (14)	0.0152 (12)	−0.0051 (10)	0.0012 (9)	−0.0048 (10)
C6	0.0215 (12)	0.0232 (14)	0.0154 (12)	−0.0066 (10)	0.0038 (9)	−0.0079 (10)
C7	0.0175 (12)	0.0231 (14)	0.0204 (13)	−0.0051 (10)	0.0033 (9)	−0.0127 (11)
C8	0.0228 (13)	0.0182 (14)	0.0237 (13)	−0.0058 (10)	0.0038 (10)	−0.0111 (11)
C9	0.0178 (12)	0.0188 (14)	0.0206 (13)	−0.0058 (10)	0.0014 (9)	−0.0102 (10)
C10	0.0185 (12)	0.0180 (14)	0.0156 (12)	−0.0052 (10)	0.0014 (9)	−0.0078 (10)
C11	0.0175 (11)	0.0192 (14)	0.0176 (12)	−0.0050 (10)	0.0026 (9)	−0.0111 (10)
C12	0.0202 (12)	0.0163 (14)	0.0190 (12)	−0.0059 (10)	−0.0007 (10)	−0.0056 (10)
C13	0.0193 (12)	0.0254 (15)	0.0173 (12)	−0.0077 (10)	0.0031 (9)	−0.0103 (10)
C14	0.0175 (12)	0.0227 (14)	0.0201 (13)	−0.0064 (10)	0.0029 (9)	−0.0126 (10)
C15	0.0216 (13)	0.0190 (14)	0.0226 (13)	−0.0046 (10)	0.0032 (10)	−0.0104 (11)
C16	0.0173 (12)	0.0172 (13)	0.0211 (13)	−0.0042 (10)	0.0036 (10)	−0.0089 (10)
C17	0.0172 (11)	0.0161 (13)	0.0155 (12)	−0.0034 (10)	0.0032 (9)	−0.0059 (10)
C18	0.0175 (11)	0.0164 (13)	0.0180 (12)	−0.0045 (10)	0.0029 (9)	−0.0089 (10)
C19	0.0183 (12)	0.0166 (13)	0.0177 (12)	−0.0029 (10)	0.0021 (9)	−0.0056 (10)
C20	0.0196 (12)	0.0237 (14)	0.0166 (12)	−0.0039 (10)	0.0005 (9)	−0.0090 (10)
C21	0.0178 (12)	0.0215 (14)	0.0219 (13)	−0.0051 (10)	0.0035 (10)	−0.0131 (11)
C22	0.0224 (13)	0.0206 (14)	0.0231 (13)	−0.0049 (11)	0.0023 (10)	−0.0109 (11)
C23	0.0176 (12)	0.0173 (14)	0.0205 (13)	−0.0041 (10)	0.0032 (9)	−0.0083 (10)
C24	0.0186 (12)	0.0170 (13)	0.0144 (12)	−0.0029 (10)	0.0012 (9)	−0.0055 (10)
C25	0.0177 (11)	0.0163 (13)	0.0173 (12)	−0.0047 (10)	0.0032 (9)	−0.0075 (10)
C26	0.0190 (12)	0.0166 (13)	0.0142 (12)	−0.0040 (10)	0.0029 (9)	−0.0031 (10)
C27	0.0199 (12)	0.0261 (15)	0.0146 (12)	−0.0072 (10)	0.0030 (9)	−0.0086 (10)
C28	0.0185 (12)	0.0229 (14)	0.0208 (13)	−0.0070 (10)	0.0049 (10)	−0.0131 (11)

### Geometric parameters (Å, °)

**Table:**

O1—C1	1.321 (3)	C2—C3	1.402 (3)
O1—H1	0.846 (10)	C3—C4	1.369 (4)
O2—C1	1.242 (3)	C3—H3A	0.9500
O3—C4	1.369 (3)	C4—C5	1.408 (3)
O3—H3	0.839 (10)	C5—C6	1.396 (4)
O4—C5	1.341 (3)	C6—C7	1.379 (4)
O4—H4	0.841 (10)	C6—H6	0.9500
O5—C8	1.320 (3)	C7—H7A	0.9500
O5—H5	0.847 (10)	C8—C9	1.457 (4)
O6—C8	1.248 (3)	C9—C14	1.394 (4)
O7—C11	1.371 (3)	C9—C10	1.405 (3)
O7—H7	0.842 (10)	C10—C11	1.368 (4)
O8—C12	1.351 (3)	C10—H10	0.9500
O8—H8	0.839 (10)	C11—C12	1.409 (4)
O9—C15	1.316 (3)	C12—C13	1.391 (4)
O9—H9	0.843 (10)	C13—C14	1.381 (4)
O10—C15	1.239 (3)	C13—H13A	0.9500
O11—C18	1.365 (3)	C14—H14	0.9500
O11—H11	0.840 (10)	C15—C16	1.466 (4)
O12—C19	1.351 (3)	C16—C21	1.394 (4)
O12—H12	0.841 (10)	C16—C17	1.397 (3)
O13—C22	1.314 (3)	C17—C18	1.380 (4)
O13—H13	0.841 (10)	C17—H17A	0.9500
O14—C22	1.247 (3)	C18—C19	1.410 (3)
O15—C25	1.368 (3)	C19—C20	1.388 (4)
O15—H15	0.844 (10)	C20—C21	1.380 (4)
O16—C26	1.350 (3)	C20—H20	0.9500
O16—H16	0.843 (10)	C21—H21	0.9500
O1w—H1w1	0.843 (10)	C22—C23	1.466 (4)
O1w—H1w2	0.842 (10)	C23—C28	1.394 (4)
O2w—H2w1	0.847 (10)	C23—C24	1.398 (4)
O2w—H2w2	0.849 (10)	C24—C25	1.375 (4)
O3w—H3w1	0.845 (10)	C24—H24A	0.9500
O3w—H3w2	0.842 (10)	C25—C26	1.404 (3)
O4w—H4w1	0.848 (10)	C26—C27	1.391 (4)
O4w—H4w2	0.855 (10)	C27—C28	1.388 (4)
C1—C2	1.454 (4)	C27—H27	0.9500
C2—C7	1.401 (4)	C28—H28	0.9500
			
C1—O1—H1	110 (2)	O8—C12—C11	116.1 (2)
C4—O3—H3	113 (2)	C13—C12—C11	118.9 (2)
C5—O4—H4	112 (3)	C14—C13—C12	120.7 (2)
C8—O5—H5	107 (3)	C14—C13—H13A	119.6
C11—O7—H7	105 (3)	C12—C13—H13A	119.6
C12—O8—H8	111 (3)	C13—C14—C9	120.2 (2)
C15—O9—H9	102 (3)	C13—C14—H14	119.9
C18—O11—H11	116 (3)	C9—C14—H14	119.9
C19—O12—H12	108 (3)	O10—C15—O9	121.9 (2)
C22—O13—H13	119 (3)	O10—C15—C16	122.2 (2)
C25—O15—H15	112 (3)	O9—C15—C16	115.9 (2)
C26—O16—H16	106 (3)	C21—C16—C17	120.1 (2)
H1w1—O1w—H1w2	109 (2)	C21—C16—C15	121.4 (2)
H2w1—O2w—H2w2	107 (2)	C17—C16—C15	118.5 (2)
H3w1—O3w—H3w2	109 (2)	C18—C17—C16	119.8 (2)
H4w1—O4w—H4w2	106 (2)	C18—C17—H17A	120.1
O2—C1—O1	121.1 (2)	C16—C17—H17A	120.1
O2—C1—C2	122.6 (2)	O11—C18—C17	119.2 (2)
O1—C1—C2	116.3 (2)	O11—C18—C19	120.7 (2)
C7—C2—C3	119.6 (2)	C17—C18—C19	120.1 (2)
C7—C2—C1	121.9 (2)	O12—C19—C20	125.2 (2)
C3—C2—C1	118.5 (2)	O12—C19—C18	115.3 (2)
C4—C3—C2	120.1 (2)	C20—C19—C18	119.4 (2)
C4—C3—H3A	120.0	C21—C20—C19	120.5 (2)
C2—C3—H3A	120.0	C21—C20—H20	119.7
O3—C4—C3	119.7 (2)	C19—C20—H20	119.7
O3—C4—C5	119.6 (2)	C20—C21—C16	120.0 (2)
C3—C4—C5	120.7 (2)	C20—C21—H21	120.0
O4—C5—C6	125.2 (2)	C16—C21—H21	120.0
O4—C5—C4	115.7 (2)	O14—C22—O13	121.7 (2)
C6—C5—C4	119.1 (2)	O14—C22—C23	122.8 (2)
C7—C6—C5	120.5 (2)	O13—C22—C23	115.5 (2)
C7—C6—H6	119.8	C28—C23—C24	119.8 (2)
C5—C6—H6	119.8	C28—C23—C22	121.7 (2)
C6—C7—C2	120.1 (2)	C24—C23—C22	118.5 (2)
C6—C7—H7A	120.0	C25—C24—C23	120.4 (2)
C2—C7—H7A	120.0	C25—C24—H24A	119.8
O6—C8—O5	121.6 (2)	C23—C24—H24A	119.8
O6—C8—C9	123.4 (2)	O15—C25—C24	123.1 (2)
O5—C8—C9	115.0 (2)	O15—C25—C26	116.9 (2)
C14—C9—C10	119.2 (2)	C24—C25—C26	120.0 (2)
C14—C9—C8	121.8 (2)	O16—C26—C27	124.9 (2)
C10—C9—C8	119.0 (2)	O16—C26—C25	115.6 (2)
C11—C10—C9	120.5 (2)	C27—C26—C25	119.5 (2)
C11—C10—H10	119.8	C28—C27—C26	120.4 (2)
C9—C10—H10	119.8	C28—C27—H27	119.8
C10—C11—O7	123.2 (2)	C26—C27—H27	119.8
C10—C11—C12	120.4 (2)	C27—C28—C23	119.9 (2)
O7—C11—C12	116.4 (2)	C27—C28—H28	120.1
O8—C12—C13	124.9 (2)	C23—C28—H28	120.1
			
O2—C1—C2—C7	−176.4 (2)	O10—C15—C16—C21	178.0 (2)
O1—C1—C2—C7	3.1 (4)	O9—C15—C16—C21	−2.1 (4)
O2—C1—C2—C3	2.5 (4)	O10—C15—C16—C17	−1.5 (4)
O1—C1—C2—C3	−177.9 (2)	O9—C15—C16—C17	178.3 (2)
C7—C2—C3—C4	0.1 (4)	C21—C16—C17—C18	0.6 (4)
C1—C2—C3—C4	−178.9 (2)	C15—C16—C17—C18	−179.9 (2)
C2—C3—C4—O3	−178.2 (2)	C16—C17—C18—O11	178.8 (2)
C2—C3—C4—C5	1.3 (4)	C16—C17—C18—C19	−1.1 (4)
O3—C4—C5—O4	−2.7 (3)	O11—C18—C19—O12	1.1 (3)
C3—C4—C5—O4	177.8 (2)	C17—C18—C19—O12	−179.0 (2)
O3—C4—C5—C6	177.8 (2)	O11—C18—C19—C20	−178.9 (2)
C3—C4—C5—C6	−1.7 (4)	C17—C18—C19—C20	1.0 (4)
O4—C5—C6—C7	−178.6 (2)	O12—C19—C20—C21	179.6 (2)
C4—C5—C6—C7	0.7 (4)	C18—C19—C20—C21	−0.4 (4)
C5—C6—C7—C2	0.6 (4)	C19—C20—C21—C16	−0.1 (4)
C3—C2—C7—C6	−1.0 (4)	C17—C16—C21—C20	0.0 (4)
C1—C2—C7—C6	178.0 (2)	C15—C16—C21—C20	−179.5 (2)
O6—C8—C9—C14	175.7 (3)	O14—C22—C23—C28	−175.5 (3)
O5—C8—C9—C14	−3.6 (4)	O13—C22—C23—C28	4.2 (4)
O6—C8—C9—C10	−3.6 (4)	O14—C22—C23—C24	3.5 (4)
O5—C8—C9—C10	177.0 (2)	O13—C22—C23—C24	−176.8 (2)
C14—C9—C10—C11	−0.1 (4)	C28—C23—C24—C25	−0.3 (4)
C8—C9—C10—C11	179.3 (2)	C22—C23—C24—C25	−179.3 (2)
C9—C10—C11—O7	179.5 (2)	C23—C24—C25—O15	−178.7 (2)
C9—C10—C11—C12	−1.1 (4)	C23—C24—C25—C26	2.1 (4)
C10—C11—C12—O8	−179.1 (2)	O15—C25—C26—O16	−1.4 (3)
O7—C11—C12—O8	0.3 (3)	C24—C25—C26—O16	177.8 (2)
C10—C11—C12—C13	1.3 (4)	O15—C25—C26—C27	178.1 (2)
O7—C11—C12—C13	−179.3 (2)	C24—C25—C26—C27	−2.6 (4)
O8—C12—C13—C14	−179.7 (2)	O16—C26—C27—C28	−179.2 (2)
C11—C12—C13—C14	−0.2 (4)	C25—C26—C27—C28	1.3 (4)
C12—C13—C14—C9	−1.1 (4)	C26—C27—C28—C23	0.6 (4)
C10—C9—C14—C13	1.2 (4)	C24—C23—C28—C27	−1.1 (4)
C8—C9—C14—C13	−178.1 (2)	C22—C23—C28—C27	177.9 (2)

### Hydrogen-bond geometry (Å, °)

**Table:**

*D*—H···*A*	*D*—H	H···*A*	*D*···*A*	*D*—H···*A*
O1—H1···O6	0.85 (1)	1.83 (1)	2.678 (3)	178 (3)
O3—H3···O1W^i^	0.84 (1)	1.99 (1)	2.803 (3)	163 (3)
O4—H4···O4W^ii^	0.84 (1)	1.86 (2)	2.674 (3)	163 (4)
O5—H5···O2	0.85 (1)	1.76 (1)	2.593 (3)	170 (4)
O7—H7···O3^iii^	0.84 (1)	1.91 (1)	2.746 (3)	172 (4)
O8—H8···O3W^iv^	0.84 (1)	1.94 (1)	2.772 (3)	171 (4)
O9—H9···O14	0.84 (1)	1.84 (2)	2.664 (3)	166 (5)
O11—H11···O3W^v^	0.84 (1)	2.02 (2)	2.824 (3)	161 (4)
O12—H12···O1W	0.84 (1)	1.92 (1)	2.760 (3)	175 (4)
O13—H13···O10	0.84 (1)	1.76 (1)	2.599 (3)	171 (5)
O15—H15···O11^vi^	0.84 (1)	1.91 (1)	2.754 (2)	178 (4)
O16—H16···O2W^vii^	0.84 (1)	1.92 (2)	2.699 (3)	153 (4)
O1W—H1W1···O2	0.84 (1)	2.36 (5)	2.920 (3)	124 (4)
O1W—H1W2···O2W^viii^	0.84 (1)	2.07 (1)	2.911 (4)	173 (7)
O2W—H2W1···O6	0.85 (1)	2.24 (1)	3.071 (3)	168 (4)
O2W—H2W2···O7^ix^	0.85 (1)	2.00 (2)	2.823 (3)	165 (4)
O3W—H3W1···O10	0.85 (1)	2.40 (4)	2.933 (3)	122 (3)
O3W—H3W2···O4W^x^	0.84 (1)	2.08 (1)	2.920 (4)	174 (6)
O4W—H4W1···O14	0.85 (1)	2.30 (3)	3.079 (3)	152 (5)
O4W—H4W2···O15^xi^	0.86 (1)	1.97 (1)	2.806 (3)	164 (4)

Symmetry codes: (i) −*x*+3, −*y*, −*z*+1; (ii) −*x*+2, −*y*+1, −*z*+1; (iii) *x*−1, *y*+1, *z*; (iv) −*x*+2, −*y*+2, −*z*; (v) −*x*+2, −*y*+1, −*z*; (vi) *x*, *y*+1, *z*; (vii) *x*−1, *y*+1, *z*−1; (viii) −*x*+3, −*y*+1, −*z*+1; (ix) −*x*+2, −*y*+2, −*z*+1; (x) −*x*+1, −*y*+2, −*z*; (xi) −*x*+1, −*y*+3, −*z*.
